# ATTACHMENT STYLE IN CHILDREN WITH CHRONIC DISEASES: A COMPREHENSIVE REVIEW

**DOI:** 10.1590/1984-0462/2020/38/2018308

**Published:** 2020-05-08

**Authors:** Virgínia Menezes Coutinho, Bianca Arruda Manchester de Queiroga, Rafaela Cristina de Souza

**Affiliations:** a Universidade Federal de Pernambuco, Recife, PE, Brazil.

**Keywords:** Object attachment, Child, Chronic disease, Apego ao objeto, Criança, Doença crônica

## Abstract

**Objective:**

To investigate how attachment style has been studied in children with chronic disease in the scientific literature, and what repercussions this attachment has on this population.

**Data sources:**

An integrative review of the literature was carried out from a survey in the LILACS, MEDLINE and SciELO databases, including original national and international publications in Portuguese, Spanish and English from 2007 to 2018, using the descriptors “*apego*” and “*criança*” in the Health Sciences Descriptors (DeCS), and “attachment” and “children” for the Medical Subject Headings (MeSH). Sixteen (16 articles) were obtained for the sample analysis.

**Data synthesis:**

The chronic diseases found in the research were neurobehavioral disorders such as attention deficit hyperactivity disorder (ADHD) and autism, childhood obesity, and chronic migraine. The predominant attachment style was insecurity, which could compromise the biopsychosocial development of the child.

**Conclusions:**

The type of attachment between child and primary caregiver may be considered a protective or risk factor for child development. Considering this premise, it is important to equip/inform families based on dialogic educational practices, in which professionals create opportunities and means for families to develop their skills and competencies, and acquire resources which meet the child’s needs. It is important that this professional helps the family to build secure bases for their child with chronic disease, understanding that the main focus for promoting child development is in the family unit.

## RESUMO

**Objetivo:**

Investigar, na literatura científica, de que forma o estilo de apego tem sido estudado nas crianças com doença crônica e as repercussões dessa vinculação nessa população.

**Fonte de dados:**

Realizou-se uma revisão integrativa da literatura por meio de um levantamento nas bases de dados Literatura Latino-Americana e do Caribe em Ciências da Saúde (LILACS), Medical Literature Analysis and Retrieval System Online (MEDLINE) e Biblioteca Eletrônica Científica Online (SciELO) incluindo publicações originais nacionais e internacionais, em português, espanhol e inglês, no período de 2007 a 2018, utilizando-se os descritores *apego* e *criança* nos Descritores em Ciências da Saúde (DeCS) e *attachment* e *children* para o Medical Subject Headings (MeSH). Foi obtida uma amostra de 16 artigos para análise.

**Síntese dos dados:**

As doenças crônicas encontradas na pesquisa foram os distúrbios neurocomportamentais, como transtorno do déficit de atenção e hiperatividade (TDAH) e autismo, obesidade infantil e enxaqueca crônica. O estilo de apego predominante foi o inseguro, o que pode comprometer o desenvolvimento biopsicossocial dessa criança.

**Conclusões:**

O tipo de vinculação entre criança e cuidador primário pode ser considerado um fator de proteção ou risco para o desenvolvimento infantil. Haja vista essa premissa, é fundamental a instrumentalização das famílias baseada em práticas educativas dialógicas, nas quais os profissionais criam oportunidades e meios para que os familiares desenvolvam suas habilidades e competências e adquiram recursos que atendam às necessidades da criança. É importante que esse profissional auxilie a família a construir bases seguras para a criança com doença crônica, entendendo que é na unidade familiar que está o principal foco de promoção do desenvolvimento infantil.

## INTRODUCTION

Chronic childhood illnesses are potentially traumatic experiences for children and their parents. During treatment and while living through the disease, families are exposed to multiple stressful events that can trigger symptoms of stress. Additionally, there is a negative impact on the psychological adjustment of the family as a whole.^1^

A child’s first years of life are of fundamental importance for his or her physical, cognitive and emotional development. In this phase, the first relationships that make up the basis for future relationships are established, highlighting the essential role of mothers and/or caregivers in responding effectively to the child’s overall needs.^2^

Bowlby’s attachment theory postulates that, in times of threat or stress, a family’s and child’s general functioning is altered, which in turn may influence the establishment of relationships, thus highlighting the biological function of intimate emotional bonds. This can be particularly relevant in the context of chronic disease, since behavioral connections at these times are even more delicate.^3^ ^,^ ^4^

After the initial assumptions of attachment theory were instituted, much research was developed. That being said, Mary Ainsworth made significant contributions to the strengthening of attachment theory. When investigating the primary relationships of many mothers and their children, Ainsworth, using an experimental method known worldwide as “strange situation,” identified different strategies of attachment behavior, which were classified into secure and insecure attachment styles, which were then subdivided into anxious and avoidant attachment styles.^5^

Caregivers who promote secure-based attachment enable children to view the outside world with positive expectations, feeling free to express feelings and thoughts, and with autonomy to explore the environment. They also show greater confidence in the attachment figure to meet their needs. In contrast, an insecure attachment style, manifested by unstable abilities, is more complex and problematic. It is the result of a relationship whose attachment figure is vulnerable and unpredictable, sometimes appearing as accessible and protective, while other times appearing as unable to provide support to the child. As a consequence, the child may have difficulties in exploring the world, insecurity, anxiety, antisocial behaviors, depression and low self-esteem.^1^ ^,^ ^6^

Corroborating Bowlby and Ainsworth, several studies highlight the association between attachment style and child development. The primary caregiver, commonly represented by the mother, is the main source of stimulus, responsible for transmitting sensory, cognitive, motor and social experiences to the child.^7^ For children with chronic illnesses, the type of chronic illness, ineffective coping with the situation, the demand of daily requirements, and certain treatments can interfere in the relationship between the mother or the primary caregiver and the child.

Due to the relevance of this relationship for the child’s biopsychosocial, cognitive and affective development, it is important that these aspects are carefully evaluated by health professionals, in a comprehensive and interdisciplinary perspective. Given the above, the need arose to investigate research that addresses attachment relationships in children with chronic illnesses and their primary attachment figure, as it is an important topic for the study of behavioral organization and the abilities children acquire in the context of this relationship.^8^

## METHOD

The present study is a comprehensive literature review whose methodological process carefully followed the following steps: identification of the theme and selection of the hypothesis or research question in order to develop the comprehensive review; establishment of inclusion and exclusion criteria for studies; categorization of studies; evaluation of studies included in the comprehensive review; interpretation of results; and presentation of the knowledge review/synthesis.^9^ ^,^ ^10^ ^,^ ^11^

This study intended to answer the following guiding question: how has attachment style been studied in children with chronic illness and what are the repercussions of this attachment in this population?

The inclusion criteria adopted for the search and selection of publications were: articles published in national and international scientific journals that addressed the theme “attachment style of children with chronic illness”; published in Portuguese, English or Spanish, in the period of 2007 to 2018; indexed in the following databases: Medical Literature Analysis and Retrieval System Online (MEDLINE), Latin American and Caribbean Literature in Health Sciences (LILACS) and Electronic Scientific Library Online (SciELO); located through the combination of the following descriptors registered in the Health Sciences Descriptors portal (*Descritores em Ciências da Saúde* - DeCS) or in the Medical Subject Headings (MeSH): “attachment” and “child, attachment” and “children”. These descriptors were combined with the Boolean operators AND and OR. This selection excluded studies that were repeated in the databases, those that were classified as a review article, those that were not presented in article format, in addition to those that did not deal with the proposed theme.

For methodological analysis of the included articles, the instrument adapted from the Critical Appraisal Skill Programme (CASP) and the Agency for Healthcare and Research and Quality’s (AHRQ) were applied.^10^ The adapted CASP includes ten items to be scored:

- Clear and justified objective.
- Appropriate methodology.
- Presentation and discussion of theoretical and methodological procedures.
- Appropriate sample selection.
- Detailed data collection.
- Relationship between researcher and respondents.
- Ethical aspects preserved.
- Rigorous and reasoned data analysis.
- Presentation and discussion of results.
- Contributions, limitations and suggestions for new research questions.

For each item, a value of zero or one was assigned, with the final result being the sum of the scores, where the maximum score is ten points. The selected articles were classified according to the scores:

- Level A: six to ten points (good methodological quality and reduced bias).
- Level B: at least five points (satisfactory methodological quality, but with an increased risk of bias).

On the other hand, the AHRQ classifies studies at seven levels, according to the amount of evidence:

- Systematic review or meta-analysis.
- Randomized clinical trials.
- Controlled trials without randomization
- Cohort and control case.
- Systematic reviews of descriptive and qualitative studies.
- The only descriptive or qualitative study.
- Opinion of authorities and/or report of specialty committees. ^12^

**Figure 1:**
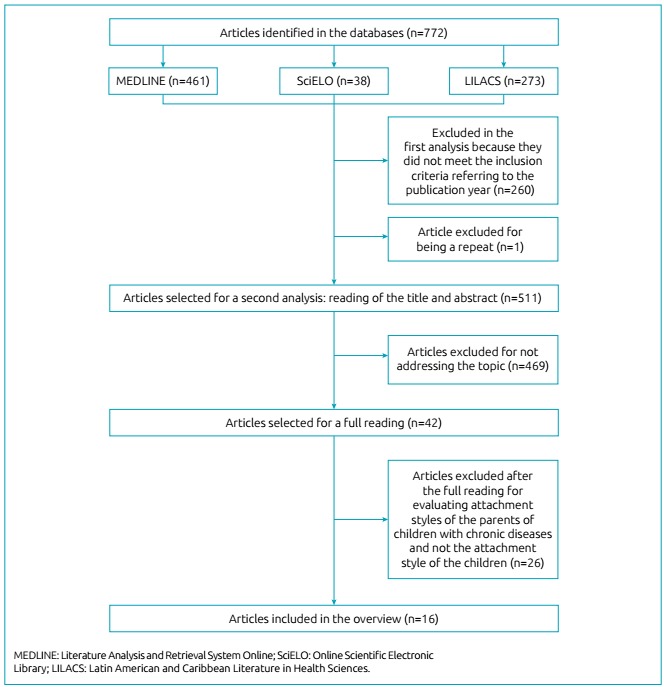
Flowchart of the search strategy for evaluating articles. Recife, PE, Brazil, 2018.

The search took place in an orderly manner, with classification in the first analysis of articles based on the period considered in the inclusion criteria, and for the second analysis, a thorough reading of the title and summary of each publication was carried out in order to verify compliance with the guiding research question, taking into account the theme and the presence of repeated articles (Figure 1). Two reviewers selected the articles as a way of ensuring greater rigor and credibility in the selection of articles and ensuring greater quality in the evaluation of the studies.

## RESULTS

In the present study, 16 articles in full were analyzed. After analyzing the selected articles (Table 1), productions from four continents were surveyed, with two subcontinents: Europe (37.5%), North America (18.75%), Asia (18.75%), South America (12.5%) and Oceania (12.5%). As for the design, 43.85% of the studies were case control, with a classification IV according to AHRQ. A total of 87.5% presented a sufficient amount of evidence A according to CASP, that is, good methodological quality and reduced bias.

**Table 1:** Characteristics of articles included in the comprehensive review, according to authors, title, country of origin, study design, Critical Appraisal Skill Program (CASP) index and Agency for Healthcare and Research and Quality’s (AHRQ) index. Recife, PE, Brazil, 2018.

Authors/year	Country of origin	Study design	CASP	AHRQ
Teague et al., 2018	Australia	Case-control	B	IV
Al-Yagon et al., 2017	Israel	Case-control	A	IV
Tarantino et al., 2017	Italy	Case-control	A	IV
Pinto et al., 2016	Portugal	Cross-sectional	A	VI
Keenan et al., 2016	Australia	Case-control	A	IV
Storebø et al., 2015	Denmark	RCT	A	II
Siller et al., 2014	United States	RCT	A	II
Grzadzinski et al., 2014	United States	Longitudinal	A	VI
Esposito et al., 2013	Italy	Case-control	A	IV
Bahrami et al., 2013	Iran	Cross-sectional	A	VI
Oppenheim et al., 2012	Israel	Cross-sectional	A	VI
Guzmán, 2012	Chile	Qualitative	A	VI
Anderson e Whitaker, 2011	United States	Cohort	A	IV
Sanini et al., 2008	Brazil	Qualitative	B	VI
Quiroga e Fanes, 2007	Spain	Case-control	A	IV
Van Ijzendoorn et al., 2007	Holland	Case-control	A	IV

RCT: Randomized clinical trial.

It was important to observe the type of chronic diseases, focusing on neurobehavioral disorders, such as attention deficit hyperactivity disorder (ADHD) and autism, in addition to childhood obesity and chronic migraines, as shown in Table 2.

**Table 2:** Characteristics of the articles included in the comprehensice review, according to authors, type of chronic disease and attachment instrument used. Recife, PE, Brazil, 2018.

Authors/year	Chronic disease	Attachment instrument
Teague et al., 2018	Autism	Child - parent relationship scale (CPRS)
Al-Yagon et al., 2017	ADHD	Hebrew version of child’s self-reported attachment security to mothers and to fathers
Tarantino et al., 2017	Migraine	Separation anxiety test
Pinto et al., 2016	Obesity	Childhood attachment inventory
Kennan et al., 2016	Autism	Security scale
Storebo et al., 2015	ADHD	Child attachment interview
Siller et al., 2014	Autism	Separation-reunion episode
Grzadzinki et al., 2014	Autism	Separation-reunion episode modified
Esposito et al., 2013	Migraine	Separation anxiety test
Bahrami et al., 2013	Obesity	Peer attachment-revised version for children
Oppenheim et al., 2012	Autism	Strange situation procedure
Guzmpan, 2012	Obesity	Child attachment interview
Anderson e Whitaker, 2011	Obesity	Toddler attachment sort
Sanini et al., 2008	Autism	Separation anxiety test
Quiroga e Fanez, 2007	ADHD	Attachment story completion task
Van Ijzendoorn et al., 2007	Autism	Strange situation procedure

ADHD: attention deficit hyperactivity disorder.

With regard to attachment style, there was a predominance of insecure attachment, especially when children with chronic diseases were compared to those in the control group, who showed typical development (according to the normality pattern) and, in general, a secure bond. According to Table 2, we can see the variety of instruments for assessing attachment style in the different studies. Although there are differences, all of these instruments considered Ainsworth’s theoretical proposals on attachment style for data interpretation.

For the purposes of analysis and discussion, after reading the articles they were grouped into categories: development of attachment in children with chronic diseases and the relationship with maternal responsiveness and sensitivity; attachment style and social skills of children with chronic illness; and interventions aimed at the family in light of the attachment style.

## DISCUSSION

### Development of attachment in children with chronic diseases and the relationship with maternal responsiveness and sensitivity

According to Ainsworth,^5^ in her theory of development, the organization of children’s attachment relationships is the cumulative result of their first interactive experiences with their caregivers. Specifically, a mother who is aware of her child’s signals is expected to interpret them accurately and respond to them promptly, in an appropriate and empathetic manner, thereby promoting the formation of a secure attachment relationship in the child.^13^

The development of attachment is a transactional relationship influenced by the characteristics of both the parent and the child. Despite the possibly different relationships that a child has with his mother versus his father, most studies have focused on attachment to the mother, as this is considered to be the main caregiver for most children. Maternal responsiveness and sensitivity are two fundamental aspects for establishing secure bonds. These dimensions include attention, communication, warmth and affection, and are associated with better levels of psychological well-being, self-esteem and self-confidence of the child.^5^

Studies selected here differ from other findings that address the influence of parenting and a child’s chronic illness on the development of attachment. Storebø et al.^14^ and Grzadzinski et al.^15^ state that biological restrictions, as well as global cognitive development, may be partly responsible for differences in attachment-related behaviors among children with autism and ADHD, believing that attachment is less secure in children with lower cognitive abilities. In contrast, Oppenheim et al.^7^ and Sanini et al.,^16^ in research with a similar sample, explain that the severity of the diagnosis and the level of cognitive and adaptive functioning of the autistic child does not reflect on their attachment. Rather it is maternal sensitivity perceptions that are responsible for this development. In the experience of a child’s chronic illness and pain associated with the diagnosis, the basis for sensitivity and maternal behavior is established, which in turn facilitates the development of a secure attachment.

Therefore, the study performed by Oppenheim et al.^7^ corroborates Ainsworth’s theory,^5^ when it said that maternal sensitivity was central in promoting attachment, characterizing sensitive mothers as those who perceive the reasons underlying their child’s behavior. As such, the theory is relevant not only for children with typical development, but also for children with chronic diseases. Thus, studies that address perception, sensitivity and attachment among children with chronic problems have implications not only for research, but also for clinical work with these children on the spectrum, and their families.

In research that dealt with neurobehavioral diseases such as autism and ADHD, it was mostly observed that both children and their mothers have a higher percentage of insecure bonds than those presented by the population with typical development.^6^ ^,^ ^7^ ^,^ ^13^ ^,^ ^14^ ^,^ ^15^ ^,^ ^16^ ^,^ ^17^ ^,^ ^18^ ^,^ ^19^ With regard to maternal representations, in addition to insecure attachment, Keenan et al.^4^ and Al-Yagon et al.^6^ reinforce the occurrence of high stress, psychological distress and anxiety experienced by these mothers. This relationship of emotional overload, often leading to overprotection and too much control, makes it difficult to develop a secure basis for the child. It interferes with the therapeutic behaviors directed at the child and can aggravate the course of the disorder or hinder the child’s recommended treatment. In view of this, children have difficulties with conflicting situations. They are unable to develop adequate solutions, which influences their ability to regulate and express their emotions, thus they often exhibit inappropriate externalizing behaviors, such as agitation and hyperactivity.^20^

Migraines were another chronic disease addressed in two studies selected in this research. Migraines are considered to be complex, and it is not fully understood how they work. In the pediatric population, headaches can be influenced by psychological symptoms and stressful life events. Furthermore, they may affect all aspects of a child’s life, such as communication, mobility, self-care, emotional competence, academic performance and cognitive functioning, coordination, sleep habits, and socialization. Considering this, they may also cause anxiety and depression.^21^ ^,^ ^22^ The relationship between headaches and attachment has been identified, and the perception of pain changes in relation to specific attachment styles, which, in turn, influence emotional regulation, as well as the tendency to seek support when exposed to a threatening situation, such as pain.^21^ ^,^ ^22^ There is evidence to suggest that attachment styles in children with migraines are significant predictors of physical and emotional distress. Empirical studies have found an association between insecure attachment and increased somatic symptoms both in adulthood and childhood. Children with insecure bonds describe their pain as more threatening and have higher levels of anxiety and depression.^23^ ^,^ ^24^

Tarantino et al. ^21^ and Esposito et al.^22^ provided the first piece of evidence regarding the role of attachment in the relationship between pain severity and psychological symptoms in children with migraines, showing that insecure attachment styles are associated with the highly frequent and severe attacks and even increased symptoms of anxiety, depression and somatization. These children show low self-esteem, are emotionally dependent on the approval of others, and are characterized by a great fear of abandonment. Driven by the desire to have their attachment needs met, these children may exhibit exaggerated nonverbal affective reactions, such as anger, fear, signs of pain or a desire for comfort, thereby provoking a more predictable response from parents.

Another chronic condition discussed in the studies in question addresses childhood obesity, which is currently considered a worldwide public health problem. In order to prevent childhood obesity and directly alter the balance between energy intake and expenditure, a healthy diet and regular physical activity are proposed. However, as interventions using these approaches are often not effective, alternative strategies need to be considered. A new measure is to help children regulate their emotions, controlling their physiological and behavioral responses. There is growing evidence linking the stress response to obesity and metabolic syndrome. High levels of stress can disrupt the functioning of the physiology of systems that affect energy balance, body weight and fat distribution.^25^

A child’s stress responses and emotional regulation begin to form in the early stages of brain development, and a secure attachment pattern is one of the best behavioral markers in regulating that child’s healthy emotions.^26^ There is a cognitive approach that addresses the influence of obese children’s mothers, specifically regarding the perception of weight and body image of their children, in which the mother is responsible for the self-perception difficulties of the obese child. Unable to establish clear parameters around him or herself, the child fails to recognize his or her own needs, resulting in a misperception of most of his or her impulses and emotions.^27^

In the studies selected in this review about the relationship between obesity and attachment, all had insecure attachments and low parental responsiveness/sensitivity.^26^ ^,^ ^27^ ^,^ ^28^ ^,^ ^29^ Pinto et al.^28^ sought to evaluate attachment strategies in obese children taking into account that the quality of the parent-child relationship plays an important role in socioemotional development, in addition to the metabolic regulation system, when a stressful event occurs. Neuroendocrine markers, including cortisol and thyroid stimulating hormone (TSH), are altered in obese people. As a result, an association was found between the avoidant insecure attachment style and higher levels of TSH and lower levels of cortisol. Corroborating Bahrami et al.,^29^ Guzmán^27^ observed an insecure bond and altered maternal responsiveness in his research. Denial and minimization of the problem, as well as carelessness, lack of a presence and negligence, including the transfer of responsibility, problems with control and containment of the child’s behavior, or even maternal overprotection, prevent the autonomy and regulation of feeding by the child and are some of the behaviors that reflect maternal interference.

In this context, regardless of the chronic health problem that the child may present, it is worth noting that a secure attachment is a protective factor that stabilizes the child and allows for better cognitive, physical and socio-affective development. These children, when establishing secure attachment with their primary care figure, perceive this relationship in a more positive way. These parents, who are considered a source of support, allow the child to develop appropriate skills in the face of conflicts and threats, and help them better regulate and express their feelings. Furthermore, it allows for children to have a greater capacity for symbolization and cognitive preparation for the situations that they face. On the other hand, an insecure attachment can be seen as a risk factor with regard these chronic conditions and the application of therapeutic measures.^20^ ^,^ ^17^

### Attachment styles and social skills of children with chronic illnesses

Al-Yagon et al.^6^ and Storebø et al.^14^ highlighted an association between ADHD and insecure attachment in children. They also demonstrated a relationship between this kind of attachment and poor social skills. Many children and adolescents with ADHD, due to issues related to their own chronic health condition, have impaired social skills, language and learning problems, and cognitive difficulties related to attention, such as: problem solving, planning, mood, and interacting with parents and teachers.^14^ The great difficulties that children with ADHD develop regarding social interaction with peers can be reinforced by negative reactions to their own disruptive behavior. The studies listed here also suggest a possible connection and influence between patterns of insecure attachment, socialization and ADHD.^6^

Like ADHD, autism also functions as a condition that influences these children’s interpersonal relationships. There is a growing appreciation for studies that address attachment style for autistic children. This interest comes with the recognition that such individuals exhibit a deficient biological development of social and emotional capacities, such as the recognition of facial emotion, social communication and reciprocity, which are central to processes that underlie the formation of attachment and relationship in child-caregiver relationships.^4^ ^,^ ^15^

In these neurobehavioral conditions, low social responsiveness, decreased shared enjoyment and difficulty in regulating affection are essential behaviors considered for the establishment of an attachment relationship.^30^ ^,^ ^31^ Therefore, the nature of these disorders, regardless of parental sensitivity, can discourage the attachment relationship between parents and children.^15^ However, despite the social and communication deficits associated with these pathologies, many studies have shown that individuals with autism and ADHD exhibit attachment behaviors similar to those of individuals with normal development or those of children with delayed development.^31^ ^,^ ^32^

Thus, although there is a biological basis for deficits in social interaction and emotional understanding, available empirical evidence suggests that children with autism and ADHD may benefit from care based on secure attachment, similarly to those with typical development.^7^ ^,^ ^17^ Insecure attachment, found in research with children with chronic problems selected here, in general influences social skills. Characteristics of this connection, such as low conflict resolution, lack of stability, irritation and loss of control, open possibilities for interventions that favor the learning of assertive behaviors for this population to deal with their daily lives in a healthier way.

### Interventions aimed at the family in the light of attachment style

Related concepts, such as emotional readiness, responsiveness and maternal insight into a child’s inner world, are associated with more secure attachment and better results in the child’s development. This is fundamental for self-confidence, self-esteem, self-control and positive relationships with others.^33^

Among the factors that can influence a mother’s and baby’s primary relationships includes incidence of disease. Parents of children with autism and ADHD, for example, face numerous challenges that parents of children with typical development do not experience, and this fact must be taken into account when considering such dyads. These families report significantly greater stress, anxiety and depression, leading to negative implications for building child attachment, presumably because these parents are less able to be consistently sensitive to their children’s needs.^4^ Given these vulnerabilities, such dyads may be at greater risk of disruption to the attachment system.^34^ ^,^ ^35^ Further exploration on the subjective experiences of the parents of a child with chronic problems is necessary. It has been documented that insecure children and adults experience higher rates of psychopathological problems than their secure counterparts. This confirms that the quality of an attachment relationship strongly predicts a variety of outcomes in a child’s life.^29^

The study by Bahrami et al.^29^ on childhood obesity highlighted the importance of attachment quality, demonstrating that an insecure style is linked to higher body weight in children. This suggests that an intervention aimed at changing the relational dynamics between parents, notably mothers, and obese children, should be included as a relevant part of childhood obesity management, since psychological problems such as depression, anxiety and symptoms related to eating disorders in children can also be associated with an insecure attachment style, thus causing damage to these children’s whole development.

Interventions in these kinds of families must be made with the objective of increasing their capacity to meet the needs of their children, including the importance of addressing the informational demands of these parents. They must use their natural environments as the intervention context (their own homes, with their own daily activities, for example). They must involve families to be active participants in this planning and intervention process, and also to promote reflection and self-assessment, considering the consequences of specific parenting strategies and choices.^13^

Studies by Siller et al.^13^ and Storebø et al.,^14^ whose purpose was to evaluate the effectiveness of an educational intervention with parents of autistic children and children with ADHD, using a control group, showed that such programs are associated with significant increases in the parents’ responsive communication. Furthermore, the children that were randomly assigned to the intervention also showed improvement in behaviors related to attachment, compared to those assigned to the control condition.^13^ ^,^ ^14^

Research on high-risk populations has linked early attachment relationships to a wide range of long-term outcomes, including children’s cognitive and language development, self-esteem, independence and school performance. This raises the hypothesis that the link between early attachment relationships and children’s long-term outcomes (eg., cognition and language) can, at least in part, be mediated by the attention given to children early on.^36^

In this context, health interventions can help both children and families to deal with the psychological and social consequences associated with chronic diseases. Interventions can improve not only the quality of interaction among these children in the various contexts in which they live - family, school, health team -, as well as in the treatment of the disease itself, through more positive responses from the child and the family to the clinical demands and demands of the disease.^37^ These actions should contribute to support reflections on the optimization of comprehensive care, by knowing how the mother is affected by the illness of her child and how she deals with this condition. Knowing the situation, resources available can be provided for the situation.^37^

Research reveals the importance of these interventions in establishing the link and development of children with chronic health problems. However, to better recognize and resolve the real needs of this population, there must be inter-professional and interdisciplinary practices, in which different areas of training are shared. It is important to be able to articulate different types of knowledge in the organization of this work, since families’ needs are heterogeneous and complex and need to be understood completely.^5^ ^,^ ^38^

Children with chronic problems may be at a much greater risk of obtaining adverse outcomes in establishing attachment and development if proper planning is not performed. Therefore, intervention programs are essential in this phase of life, as they can minimize maternal psychological suffering caused by the difficulty of raising a child that is often perceived as incomplete. Intervention programs can assist parents in the management of these situations and offer psychological support.^20^ It is necessary for health professionals to be attentive to aspects that transcend the medical treatment of a child’s disease, since, without a comprehensive view about the child’s growth, and their relationships with the significant figures in their life, the success of the treatment can be compromised. Therefore, it is essential that actions are also based on this primary caregiver, expanding the view of family relationships that directly or indirectly affect the health-disease process^37^ of the child. As such, it is essential to properly equip families, using educational dialogic practices through which professionals create opportunities and means for all caregivers to observe their skills and abilities and acquire resources that meet the needs of the child, while reinforcing sensitivity and responsiveness.

This review has some limitations, such as the predominant methodological design, which does not guarantee a cause and effect relationship for the observed associations. Additionally, there are the non-probabilistic samples, which also exhibit inferential limitations. It is worth noting the small number of studies available on the subject in South America, which restricts the extrapolation of some findings.

A small variety of chronic diseases was observed with regard to this theme, preventing a more reliable generalization of the results. Furthermore, there were few studies that evaluated the association between the child’s attachment style and his or her cognitive development, since an important repercussion of an insecure attachment is the negative impact on this kind of development. It is also worth noting that the instruments that assess attachment have not yet been validated in Brazil. They have only undergone an adaptation, as in the study by Sanini et al.^16^ Brazilian surveys normally use the “separation anxiety test” (SAT) to assess addiction. They justify its use by saying it is a projective test that adapts to the Brazilian reality and to the population it intends to study.^39^

However, even with all the limitations presented here, the present study still contributes to the theory of attachment, because, in addition to expanding its scope in terms of the populations studied, it also opens up space for new initiatives to be developed.

## FINAL CONSIDERATIONS

The authors hope that the present study can contribute to new research in the field, mainly in studies with other chronic diseases in children, since attachment relationships in this population are still concentrated in certain areas and there is a small number of studies on them. Knowing that the relationship between parents and children is linked to the development of these children is an important tool for the construction of a care plan. The specifics of each situation must be considered for planning strategies aimed at promoting health in this group. It is important that health professionals assist in the construction of secure attachments with regard to children with chronic illness, because the best way to promote child development is in the family unit.
